# Dual recognition of H3K4me3 and H3K27me3 by a plant histone reader SHL

**DOI:** 10.1038/s41467-018-04836-y

**Published:** 2018-06-21

**Authors:** Shuiming Qian, Xinchen Lv, Ray N. Scheid, Li Lu, Zhenlin Yang, Wei Chen, Rui Liu, Melissa D. Boersma, John M. Denu, Xuehua Zhong, Jiamu Du

**Affiliations:** 1 Laboratory of Genetics, University of Wisconsin-Madison, Madison, WI 53706 USA; 2 Wisconsin Institute for Discovery, University of Wisconsin-Madison, Madison, WI 53706 USA; 3 National Key Laboratory of Plant Molecular Genetics, CAS Center for Excellence in Molecular Plant Sciences, Shanghai Center for Plant Stress Biology, Shanghai Institutes for Biological Sciences, Chinese Academy of Sciences, Shanghai, 201602 China; 4 University of Chinese Academy of Sciences, Beijing, 100049 China; 5 Department of Biomolecular Chemistry, University of Wisconsin-Madison, Madison, WI 53706 USA; 6 Morgridge Institute for Research, Madison, WI 53706 USA

## Abstract

The ability of a cell to dynamically switch its chromatin between different functional states constitutes a key mechanism regulating gene expression. Histone mark “readers” display distinct binding specificity to different histone modifications and play critical roles in regulating chromatin states. Here, we show a plant-specific histone reader SHORT LIFE (SHL) capable of recognizing both H3K27me3 and H3K4me3 via its bromo-adjacent homology (BAH) and plant homeodomain (PHD) domains, respectively. Detailed biochemical and structural studies suggest a binding mechanism that is mutually exclusive for either H3K4me3 or H3K27me3. Furthermore, we show a genome-wide co-localization of SHL with H3K27me3 and H3K4me3, and that BAH-H3K27me3 and PHD-H3K4me3 interactions are important for SHL-mediated floral repression. Together, our study establishes BAH-PHD cassette as a dual histone methyl-lysine binding module that is distinct from others in recognizing both active and repressive histone marks.

## Abstract (web summary)

Histone mark reader proteins bind to particular histone modifications and regulate chromatin state. Here, Qian et al. show that the SHORT LIFE reader has a unique ability to recognize both activating and repressive histone marks and that these interactions enable SHORT LIFE to repress flowering in plants.

## Introduction

Epigenetic modification is an important gene regulatory mechanism conserved in plants, animals, and fungi and impacts many biological processes including genome defense, imprinting, proper development and physiology, and environmental responses^1–7^. Aberrant modification patterns are associated with a wide variety of developmental defects and diseases^8–13^. One major type of epigenetic modification is DNA methylation, the addition of a methyl group to DNA cytosine bases. Post-translational modification (PTM) to histone proteins is another epigenetic mark and forms complex interconnections with DNA methylation^4, 14–17^. Epigenetic modifications are added, removed, and recognized by specific proteins and/or protein domains named “writer”, “eraser”, and “reader”, respectively, in which the “reader” modules can serve as the molecular interpreters to translate the complex PTM codes to certain functional outputs and to transmit these signals to downstream effectors^18–20^.

Emerging evidence suggests that chromatin reader domains exhibit distinct binding specificity to different histone PTMs and this specific PTM recognition contributes to either repressive or active chromatin states to modulate gene expression. For example, bromodomain functions mainly as acetyl-lysine recognition motifs^21^. Plant homeodomain (PHD) fingers are diverse chromatin-binding modules for various histone marks, including methylated, unmethylated, and acetylated lysine residues with different sequence contexts^22^. Chromodomain family proteins display strong binding preference for methylated histone lysine residues^23^. Bromo-adjacent homology (BAH) is an evolutionarily conserved binding module known to recognize distinct histone modifications^24^. Interestingly, many reader proteins contain multiple histone recognition domains that often exist in tandem and function in multivalent chromatin binding to elicit high specificity and avidity to the appropriate epigenetic landscapes^25, 26^.

Currently, the majority of multivalent reader proteins engages similar PTM states: either active or repressive histone marks. For example, human BPTF recognizes the active histone marks H3K4me3 and H4K16ac by its PHD and bromo domains, respectively^27, 28^. Similarly, Arabidopsis CHROMOMETHYLASE3 (CMT3) contains a BAH domain and a chromodomain, both interacting with the repressive mark H3K9me2^29^. Interestingly, the PHD-bromo cassette of TRIM33 can recognize both the silencing mark H3K9me3 and the active mark H3K18ac^30^. In plants, the histone mark readers are generally similar to their animal counterparts but with some plant-specific features. Plant histone reader proteins often have unique reader domain combinations and different reader-mark pairs^31, 32^.

In Arabidopsis, the BAH and PHD dual domain-containing proteins SHORT LIFE (SHL) and EARLY BOLTING IN SHORT DAYS (EBS) function in chromatin-mediated floral repression and seed dormancy^33–37^. Despite the similar domain structures, the two paralogs SHL and EBS adopt different mechanisms regulating flowering by repressing distinct floral genes^33^. Genetically, while EBS regulates the expression of a floral integrator FLOWERING LOCUS T (FT), SHL has been shown to repress another floral integrator SUPPRESSION OF OVEREXPRESSION OF CO1 (SOC1) to inhibit flowering^33, 35^. Despite the repressive role of SHL, the molecular mechanism underlying SHL floral repression remains largely unexplored. Here, our biochemical and X-ray structural studies show that SHL is capable of recognizing an active histone mark H3K4me3 and a repressive mark H3K27me3 independently via its PHD and BAH domain, respectively. Chromatin immunoprecipitation followed by sequencing shows that a subset of SHL-bound genes was co-marked with H3K4me3 and H3K27me3. Mutations abolishing BAH-H3K27me3 and PHD-H3K4me3 binding cause a failure of SHL in complementing the early *shl* flowering phenotype, suggesting that SHL binding to H3K27me3 and H3K4me3 is functional in vivo. Collectively, we establish a single protein-mediated dual histone mark recognition and provide insights into establishing and maintaining proper chromatin landscapes.

## Results

### SHL is an H3K27me3 reader that can also bind H3K4me3

**Fig. 1:**
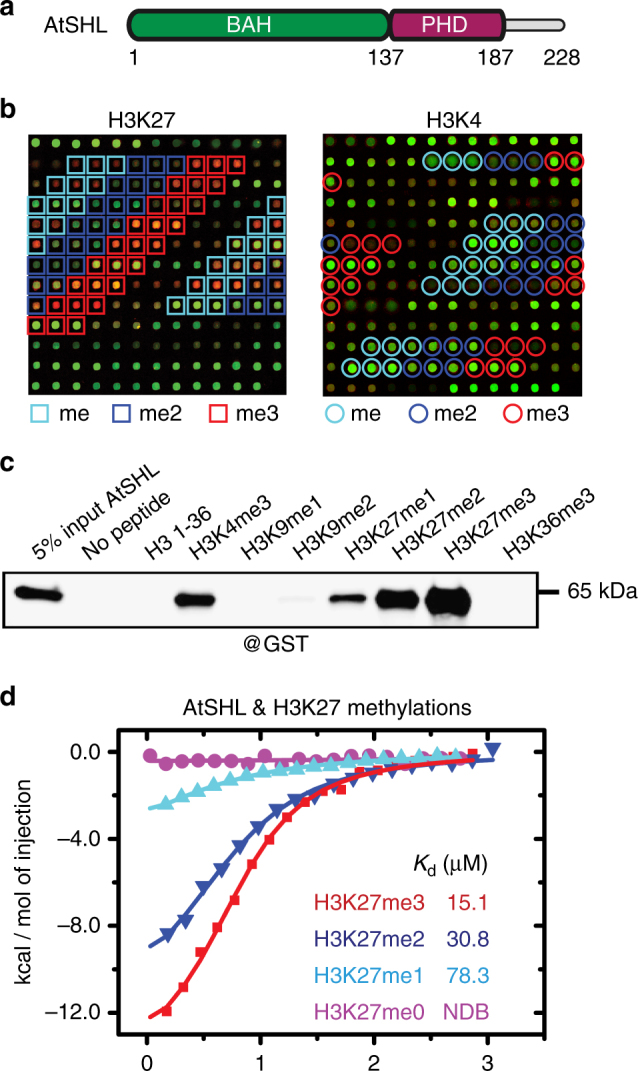
SHL binds H3K27me3 and H3K4me3. **a** The domain architecture of Arabidopsis SHL (AtSHL). BAH, bromo-adjacent homology; PHD, plant homeodomain. **b** Systematic profiling of histone binding preferences of full-length AtSHL on a histone peptide microarray. Representative array images showing that SHL preferentially binds to H3K27me3 and H3K4me3. Light blue, dark blue, and red squares represent the locations of mono, di, and trimethylated H3K27-containing peptides on the array, respectively. Light blue, dark blue, and red circles represent the locations of mono, di, and trimethylated H3K4-containing peptides on the array, respectively. **c** Immunoblotting analysis of histone peptide pulldown with full-length AtSHL. **d** ITC binding curves for complex formation between AtSHL and different H3K27 methylated peptides. NDB, no detectable binding

Although SHL and EBS are paralogs and possess similar domain architecture of an N-terminal BAH domain and C-terminal PHD finger, they can be classified into two independent clades based on the phylogenic analysis (Fig. 1a and Supplementary Fig.1a), and in addition, have distinct C-termini^33^. PHD of Arabidopsis SHL (AtSHL) was previously shown to bind to H3K4me2/3 in vitro by pull down assays^33^. Prior studies showed that BAH domains are also histone mark readers^22, 24^. To explore the function of BAH and further identify the histone binding mode of SHL, we purified the full-length recombinant AtSHL protein and screened it on a histone peptide microarray^38^. We found that AtSHL bound H3K4me3 (Supplementary Fig. 1b and Supplementary Data 1), consistent with previous findings^33^. Surprisingly, AtSHL also displayed a strong binding affinity to a repressive histone mark H3K27me3 (Fig. 1b, Supplementary Fig. 1c–d, and Supplementary Data 1). Our in vitro pulldown assay using biotinylated histone peptides further confirmed that AtSHL is capable of binding to H3K27me3 and H3K4me3 (Fig. 1c).

To quantify the binding affinity, we performed an isothermal titration calorimetry (ITC) assay and found a dissociation constant (*K*_d_) of 15.1 µM between AtSHL and H3K27me3 peptides (Fig. 1d). The binding affinity dropped to 30.8 µM and 78.3 µM for H3K27me2 and H3K27me1, respectively, and no binding was observed for the unmodified H3K27 peptide (Fig. 1d). Taken together, these results suggest that AtSHL is a histone reader protein capable of recognizing both H3K4me3 and H3K27me3 histone marks.

### Recognition of H3K4me3 by PHD finger of SHL

**Fig. 2:**
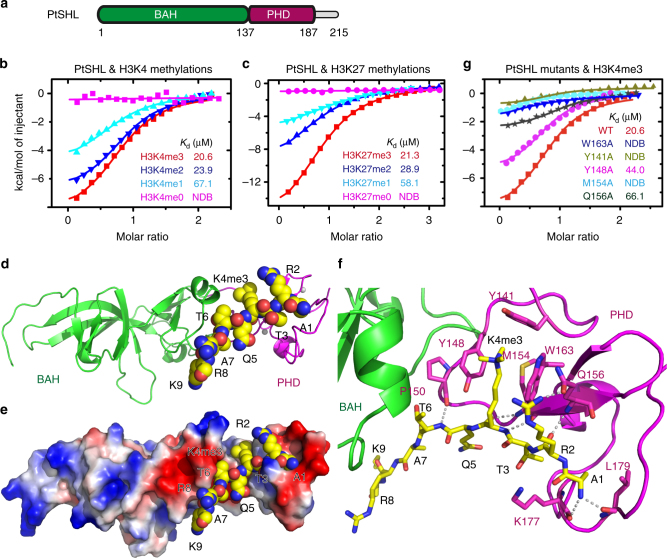
Structure of PtSHL in complex with H3K4me3. **a** The domain architecture of *Populus trichocarpa* SHL (PtSHL). BAH, bromo-adjacent homology; PHD, plant homeodomain. **b, c** ITC binding curves for the complex formation between PtSHL and different H3K4 (b) and H3K27 (c) methylated peptides. **d** Overall structure of PtSHL-H3K4me3 complex with the BAH and PHD domains colored in green and magenta, respectively. The peptides and zinc ions are shown as spacing fill model and silver balls, respectively. **e** Electrostatic surface view of the PtSHL in complex with H3K4me3. **f** The detailed interactions between PtSHL and H3K4me3 with the peptide shown in stick model. The hydrogen bonds are highlighted by dashed lines. **g** ITC binding curves show that the mutations of key residues critical for H3K4me3 binding significantly decrease the binding affinity to the H3K4me3 peptide. NDB, no detectable binding

To gain molecular insights into the reader function of SHL, we performed structural studies on SHL. Despite efforts on AtSHL crystallization, we were unable to obtain AtSHL crystal either in a free form or in complex with histone peptides. In contrast, the SHL from *Populus trichocarpa* (PtSHL), which has similar PHD and BAH domains (Fig. 2a) and shows similar H3K4me3 and H3K27me3-binding affinities (Fig. 2b, c), successfully yielded crystals. We determined the crystal structure of PtSHL-H3(1–15)K4me3 complex at 2.8 Å resolution (Fig. 2d and Table 1). The peptide adopts an extended conformation and fits into a negatively charged surface cleft of the PHD finger of PtSHL (Fig. 2d, e and Supplementary Fig. 2a). The amino protons of H3A1 form two hydrogen bonds with the PHD finger thereby anchoring the H3 N-terminus (Fig. 2f).

**Table 1:** Data collection and refinement statistics

	PtSHL-H3K4me3	PtSHL-H3K27me3
**Data collection**		
PDB code	5ZNP	5ZNR
Beamline	SSRF-BL19U1	SSRF-BL19U1
Space group	*P*2_1_2_1_2_1_	*P*2_1_
Wavelength (Å)	0.9792	0.9792
Cell dimensions		
*a*, *b*, *c* (Å)	44.3, 91.9, 114.4	43.7, 110.3, 58.6
α, β, γ (°)	90, 90, 90	90, 96.6, 90
Resolution (Å)	50.0–2.8 (2.90–2.80)^a^	50.0–3.2 (3.31–3.20)
*R*_merge_	0.136 (0.770)	0.190 (0.786)
*I*/ σ*I*	11.2 (1.6)	5.4 (1.2)
Completeness (%)	99.4 (99.6)	99.7 (99.8)
Redundancy	4.3 (4.2)	3.8 (3.9)
**Refinement**		
No. reflections	12,004	9,089
*R*_work_/*R*_free_	0.250/0.293	0.206/0.232
No. atoms	3,151	3,165
Protein	2,983	2,900
Peptide/Zn^2+^	142/4	216/4
Water/SO_4_^2−^	22/0	0/45
*B*-factors (Å^2^)	73.5	66.0
Protein	72.7	65.7
Peptide/Zn^2+^	92.8/108.6	65.9/79.2
Water/SO_4_^2−^	54.9/ –	–/85.0
RMS deviations		
Bond lengths (Å)	0.005	0.010
Bond angles (°)	1.066	1.156

^a^Highest-resolution shell is shown in parentheses

The trimethyllysine of H3K4me3 inserts into and is specifically recognized by a canonical aromatic cage formed by Tyr141, Tyr148, and Trp163, thereby resembling other reported H3K4me3-reading PHD fingers^22, 39^ (Fig. 2f). H3R2, H3K4me3, and H3T6 form additional hydrogen-bonding interactions with Gln156, Met154, and Pro150, respectively (Fig. 2f). Single amino acid substitution of the key residues involving PtSHL-H3K4me3 interaction either abolished or greatly reduced the binding to H3K4me3 (Fig. 2g). Besides the PHD finger, the BAH domain has direct but not sequence-specific contacts with H3T6 to H3K9 (Fig. 2d–f).

### Recognition of H3K27me3 by BAH domain of SHL

**Fig. 3:**
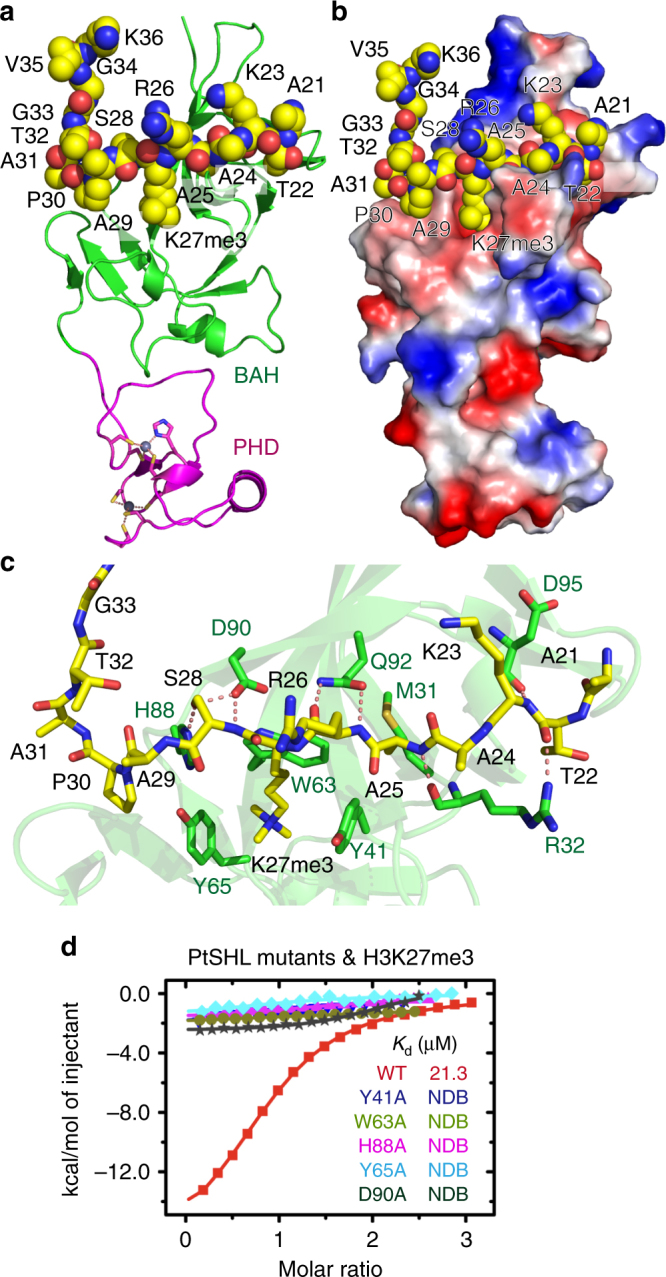
Structure of PtSHL in complex with H3K27me3. **a** Overall structure of PtSHL in complex with H3K27me3 peptide. The bromo-adjacent homology (BAH) and plant homeodomain (PHD) domains are colored in green and magenta, respectively. The peptide is shown in space filling representation. **b** Electrostatic surface view of the PtSHL in complex with H3K27me3. **c** Details of the specific recognition of H3K27me3 by an aromatic cage of BAH. The hydrogen-bonding interactions are highlighted with dashed red lines. **d** ITC binding curves between PtSHL mutants and H3K27me3 peptides reveal that the mutations of key H3K27me3 recognition residues impair the binding. NDB, no detectable binding

We further determined the structure of the PtSHL-H3(20–36)K27me3 complex at 3.2 Å resolution (Fig. 3a and Table 1). The peptide adopts a right angle-like conformation with a sharp turn at H3A31 and is bound within a negatively charged surface cleft on the BAH domain (Fig. 3a, b and Supplementary Fig. 2b). In detail, the interaction features the recognition of the H3K27me3 trimethyllysine by a classical aromatic cage formed by Tyr41, Trp63, and Tyr65 (Fig. 3c**)**, similar to other canonical methyl-lysine readers^39^. In addition, Tyr41, Trp63, and Met31 form a shallow hydrophobic pocket to accommodate the methyl group of H3A25 (Fig. 3c).

Interestingly, the side chain hydroxyl group of H3S28 is anchored and fixed between PtSHL His88 and Asp90 and forms two hydrogen bonds with them (Fig. 3c). The interaction between H3S28 and PtSHL His88 fixes the side chain rotamer of His88, locking the conformation of the imidazole ring of His88 in a parallel alignment with the prolyl ring of H3P30 with the alignment stabilized by hydrophobic and CH–π interactions (Fig. 3c). Although H3K9 and H3K27 share the ARKS consensus motif, H3P30 at the n + 3 position of H3K27me3 (but not H3G12 at the n + 3 position of H3K9me3) can be specifically recognized by PtSHL, suggesting a sequence-specific recognition mechanism. Mutations of His88 (H88A) or Asp90 (D90A) involved in H3P30 recognition as well as mutations of the aromatic residues responsible for H3K27me3 recognition (Y41A, W63A, Y65A), abolished the binding of PtSHL with H3K27me3 (Fig. 3d). In addition, Arg32, Asp90, Gln92, and Asp95 form several hydrogen bonds with the main chain of the bound H3K27me3 peptide (Fig. 3c).

Notably, all key residues involved in H3K4me3 and H3K27me3 binding are conserved in SHL proteins from different species (Supplementary Fig. 3), indicating conserved across species H3K4me3binding and H3K27me3-binding functions of SHL PHD and BAH domains, respectively.

### Recognition of H3K4me3 and H3K27me3 by SHL are independent

**Fig. 4:**
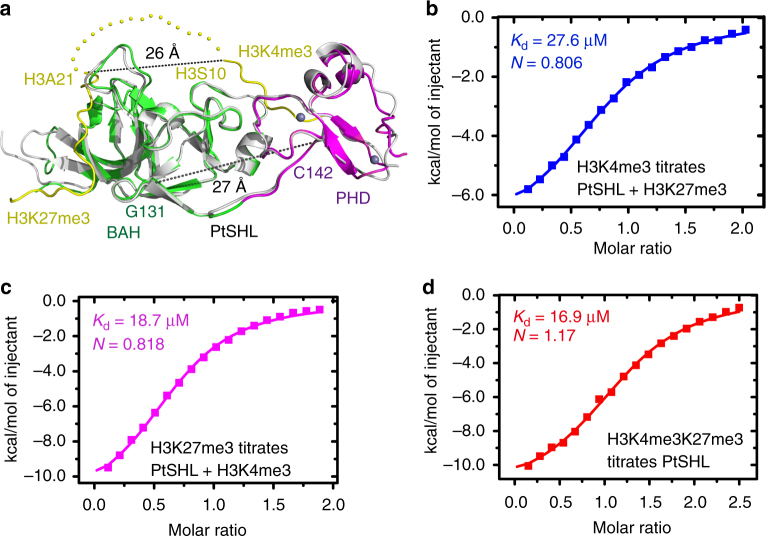
H3K27me3-BAH and H3K4me3-PHD finger binding are independent of each other. **a** A superposition of PtSHL-H3K27me3 (in colored scheme) and PtSHL-H3K4me3 (in silver) complexes. The distance between H3S10 to H3A21 and the distance between Gly131 and Cys142 as a reference are highlighted as dashed lines. **b** The ITC binding curve of 2-fold excess H3(20-36)K27me3 pre-saturated PtSHL titrated by H3(1-15)K4me3 peptides. **c** The ITC binding curve of 2-fold excess H3(1-15)K4me3 pre-saturated PtSHL titrated by H3(20-36)K27me3 peptides. **d** The binding between PtSHL and H3(1-35)K4me3K27me3 peptide yielded a binding affinity nearly identical as PtSHL-H3K4me3 and PtSHL-H3K27me3 and with an *N*-value of 1.17

Our biochemical and structural data have confirmed that SHL can specifically recognize both the active mark H3K4me3 and repressive mark H3K27me3. The superposition of the two complexes yields an RMSD of 0.94 Å, suggesting a nearly identical overall structures (Fig. 4a). The distance between H3S10 (the last observed residue of the H3K4me3 peptide) and H3A21 (the first observed residue of the H3K27me3 peptide) is 26 Å (Fig. 4a). As a reference, a 10-residue gap between Gly131 and Cys142 that is connected by an extended straight loop also requires 27 Å (Fig. 4a). Considering the parallel directionalities of the two peptides that require two additional turns to connect them, it is quite challenging for SHL to simultaneously capture both H3K4me3 and H3K27me3 marks with an H3K4me3K27me3 dual mark containing peptide structurally.

To further investigate the binding mechanism of H3K4me3 and H3K27me3 by the two domains of SHL, we performed competitive binding assays. The binding between 2-fold excess H3(20–36)K27me3 peptide pre-saturated PtSHL and H3(1–15)K4me3 peptide yielded a binding affinity of 27.6 μM (Fig. 4b), similar to the binding affinity of 20.6 μM between PtSHL and H3(1–15)K4me3 peptide (Fig. 2b). Moreover, the binding between 2-fold excess H3(1–15)K4me3 pre-saturated PtSHL and H3(20–36)K27me3 yielded a binding affinity of 18.7 μM (Fig. 4c), similar to the 21.3 μM binding between PtSHL and H3K27me3 (Fig. 2c), suggesting that the recognition of H3K4me3 by the PHD finger and the recognition of H3K27me3 by the BAH domain are independent of each other.

Next, we investigated if a histone tail bearing both H3K4me3 and H3K27me3 marks can be combinatorially recognized by SHL. The ITC binding between PtSHL and an H3(1–35)K4me3K27me3 peptide yielded a binding affinity of 16.9 μM and a binding *N*-value of 1.17 (Fig. 4d), indicating a 1:1 binding ratio of PtSHL and doubly methylated H3(1–35)K4me3K27me3 peptide. This 1:1 stoichiometry suggested that only one H3 tail can be captured by SHL and the distance between H3K4me3 and H3K27me3 sites of a single histone tail is insufficient to accommodate two SHL molecules. The 1:1 stoichiometry coupled with an observation that there is no significant binding affinity difference between singly and doubly modified H3K4me3 and H3K27me3 peptides (Fig. 4d), suggest that the PHD-H3K4me3 and the BAH-H3K27me3 binding are independent events and mutually exclusive.

### Co-localization of SHL with H3K27me3 and H3K4me3 in vivo

**Fig. 5:**
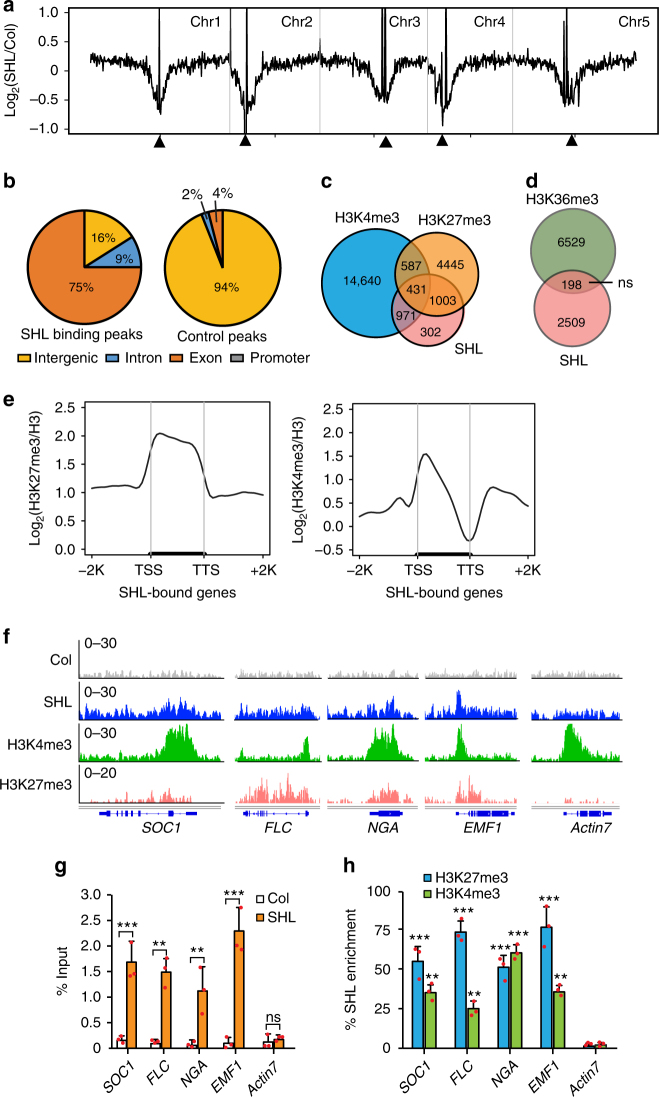
SHL co-localizes with H3K4me3 and H3K27me3 in the genome. **a** Chromosomal views show that SHL is highly enriched in gene-rich euchromatic regions. *Y*-axis represents the Log_2_ value of ChIP-seq reads normalized to wild-type Col. The Chr1, Chr2, Chr3, Chr4, and Chr5 represent the five Arabidopsis chromosomes. Black triangle indicates the location of the centromere. **b** Genomic distribution of SHL ChIP-seq peaks. Control peaks are the same numbers of regions as SHL-bound peaks randomly selected from the genome. **c** Venn diagram showing the overlap of SHL-, H3K4me3-, and H3K27me3-enriched genes. **d** Venn diagram showing the overlap of SHL-bound genes with H3K36me3-marked genes. The *P* value is based on the hypergeometric test. ns, not significant. **e** Metaplots of H3K27me3 and H3K4me3 levels over the transcription unit of SHL-bound genes. *Y*-axis represents the levels of histone modifications normalized with H3. TSS, transcription start site; TTS, transcription termination site; -2K, 2kb upstream of TSS; +2K, 2kb downstream of TTS. **f** Genome-browser view of normalized ChIP-seq peaks at representative loci. **g** Relative enrichment of SHL at the indicated loci in (**f**) calculated as the percent of input materials. **h** Sequential ChIP-qPCR showing the H3K4me3 and H3K27me3 enrichment relative to SHL at the indicated loci in (**f**). *Actin7* serves as a control locus. All error bars represent mean ± SD from three independent experiments. Red dots denote the individual data points. Student’s *t*-test was used to calculated the *P* value. *P < 0.05; **P < 0.01; ***P < 0.001; ns, not significant

The ability of a single SHL protein to recognize both H3K4me3 and H3K27me3 in vitro promoted us to determine whether SHL is important for the steady-state levels of H3K4me3 and H3K27me3. We performed immunoblotting experiments and found no noticeable difference of H3K4me3 and H3K27me3 levels in *shl* when compared to the wild-type (Supplementary Fig 4a). We next determined whether SHL co-localizes with H3K4me3 and H3K27me3 in vivo. We generated Arabidopsis transgenic plants expressing SHL-3xFLAG driven by its native promoter (pSHL::SHL-3xFLAG, abridged as SHL-FLAG) and performed chromatin immunoprecipitation coupled with high-throughput sequencing (ChIP-seq). The genome-wide distribution analysis across the five Arabidopsis chromosomes revealed that SHL is specifically enriched in euchromatic arms (Fig. 5a). We identified a total of 2629 SHL peaks (*P* < 0.001) distributed in 2707 genes (Fig. 5b and Supplementary Data 2). The peaks were highly enriched in exons (75%) and introns (9%) compared to the randomly selected control peaks (Fig. 5b). Further analysis revealed a co-occupancy of SHL (~ 89% SHL-bound genes) with either H3K27me3 or H3K4me3. Appropriately 53% (1434 out of 2707) and 38% (1018 genes) of SHL-bound genes were co-marked with H3K27me3 and H3K4me3, respectively (Fig. 5c). Among them, 431 SHL-enriched genes were overlapped with both marks (Fig. 5c). Next, we performed Venn diagram analysis to overlap the SHL-associated genes with another active histone mark and found that only 198 genes (7%) were overlapped with H3K36me3-marked genes (Fig. 5d). Consistently, we noted a significant enrichment of H3K27me3 and to a less extent of H3K4me3 at the SHL-bound genes (Fig. 5e).

To provide additional evidence, we performed a sequential ChIP assay in which SHL was first enriched with an initial ChIP and the co-enrichment of H3K4me3 and H3K27me3 was determined by a re-ChIP either with an H3K4me3 or H3K27me3 antibody. We examined four loci that were bound by SHL and co-marked with H3K4me3 and H3K27me3 (Fig. 5f) and found a significant co-enrichment of H3K27me3 and H3K4me3 with SHL compared to wild-type Col (Fig. 5g, h). Collectively, these results showed that SHL is associated with the regions co-enriched with H3K4me3 and H3K27me3 in vivo.

### BAH and PHD domains are important for SHL function in vivo

**Fig. 6:**
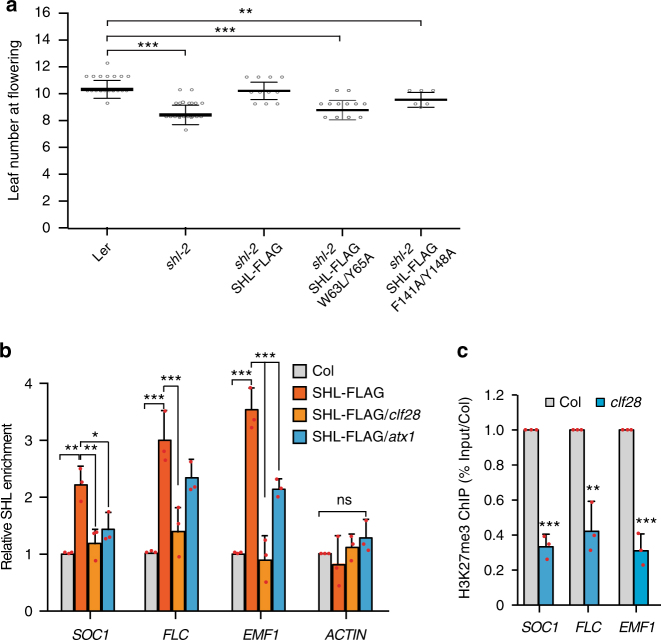
BAH and PHD are important for SHL function in vivo. **a** Flowering time analysis of long-day grown plants of wild-type L*er*, *shl-2, shl-2* mutants transformed with SHL-FLAG constructs encoding wild-type SHL (SHL-FLAG), H3K27me3 (SHL-FLAG W63L/Y65A) or H3K4me3 (SHL-FLAG F141A/Y148A) binding defective mutants. Each white circle of the SHL-FLAG, SHL-FLAG W63L/Y65A, and SHL-FLAG F141A/Y148A represents individual T1 transgenic plant. Black horizontal lines are mean and the error bars represent mean ± SD from the number of plants (range of 5-20 plants) counted for each line. **b** Relative SHL enrichment in wild-type, *clf28*, and *atx1* mutants. **c** ChIP-qPCR of relative H3K27me3 levels in Col and *clf28* at three loci. Error bars represent mean ± SD from three independent experiments. Red dots denote the individual data points. Student’s *t*-test was used to calculated the *P* value. *P < 0.05; **P < 0.01; ***P < 0.001; ns, not significant

The ability of SHL to bind to H3K27me3 and H3K4me3 in vitro and their co-localization in vivo promote us to investigate the functional significance of SHL-H3K27me3 and SHL-H3K4me3 binding. We generated point mutations within the aromatic cage of BAH (W63L/Y65A) and PHD (F141A/Y148A) that abolished SHL binding to H3K27me3 and H3K4me3, respectively (Figs. 2g and 3d). These point mutations were engineered into an epitope-tagged SHL construct (pSHL::SHL-3xFLAG) and transformed into a *shl-2* mutant background. The ability of transgenic plants carrying specific point mutations to restore *shl-2* induced early flowering phenotype^33^ was assayed by counting the leaf number at the flowering stage. As expected, addition of a wild-type AtSHL transgene rescued the early flowering phenotype caused by *shl-2* (Fig. 6a). We found that the H3K27me3-binding defective mutant (SHL W63L/Y65A) was unable to rescue the early *shl-2* flowering phenotype and H3K4me3-binding defective mutant (SHL F141A/Y148A) only partially rescued the early *shl-2* flowering phenotype (Fig. 6a). To rule out the possibility that the failure in full complementation is due to the instability of the mutant proteins, we assessed the protein levels of SHL W63L/Y65A and SHL F141A/Y148A and found that mutant version of SHL has similar levels as the wild-type SHL (Supplementary Fig. 4b). These results suggest a critical role of BAH-H3K27me3 and PHD-H3K4me3 binding for SHL function in vivo.

Next, we asked whether SHL chromatin association requires the pre-existence of H3K27me3 or H3K4me3 in vivo. To address this question, we determined the chromatin association of SHL in a loss-of-function H3K27me3 methyltransferase CURLY LEAF (CLF)^40^ mutant as well as an H3K4me3 methyltransferase ARABIDOPSIS TRITHORAX1 (ATX1) mutant^41, 42^. We found that SHL chromatin association at several target genes was significantly decreased along with reduced H3K27me3 levels in the *clf28* mutant (Fig. 6b, c), suggesting that SHL chromatin occupancy is dependent on H3K27me3 at these loci. We also noted a moderate reduction of SHL enrichment in *atx1* mutant (Fig. 6b), despite having similar SHL levels as wild-type (Supplementary Fig. 4c). The partial reduction is likely due to the presence of other ATX members or ATX-related proteins redundantly required for H3K4me3 levels^41–43^.

## Discussion

An epigenetic paradox is the ability to maintain a stable epigenetic state for many cellular generations, yet also to be flexibly switched to an alternative chromatin state at any stage. Specific recognition of histone marks by “reader” proteins constitutes an important mechanism in establishing and maintaining proper epigenetic landscapes. In this study, we identified SHL as a dual histone methyl-lysine reader that is distinct from other reader proteins in recognizing both H3K4me3 and H3K27me3, two well-known histone marks with opposing roles in balancing chromatin and transcriptional states^44, 45^. Our biochemical and structural analyses provide the molecular basis of H3K27me3 recognition by BAH and H3K4me3 by PHD finger. This represents an example of a dual recognition mode for two antagonistic histone methylation marks mediated by one single protein. Previously, only multi-protein complexes were known to display H3K4me3-H3K27me3 crosstalk^17, 44–46^. Thus, the BAH-PHD dual binding cassette may represent a regulatory circuit to mediate the chromatin switching between an active and repressive state.

The BAH domain has been extensively investigated both functionally and structurally as a histone reader domain^24^, with the histone marks recognized by BAH domains representing a diversified set. Maize ZMET2, mouse ORC1, and Arabidopsis ORC1b utilize their BAH domains to recognize H3K9me2, H4K20me2, and unmodified H3, respectively (Supplementary Fig. 5a–c)^29, 47, 48^. Here, we revealed that SHL BAH is engaged in H3K27me3 recognition, similar to the BAH domain of BAHD1 protein^49^. The methyl-lysine binding BAH domains employ a canonical aromatic cage to accommodate the bound methyl-lysine, indicating a conserved methyl-lysine recognition function of the BAH domain (Supplementary Fig. 5a, b, d). Additionally, different flanking sequences function as filters to endow the BAH domains with sequence-specificity. The Arabidopsis ORC1b BAH domain together with a PHD finger combinatorially recognize unmodified H3 using a different interface (Supplementary Fig. 5c)^47^, suggesting a distinct histone binding interface other than the aromatic cage.

Our competitive ITC binding experiments reveal that bindings between H3K4me3-PHD finger and H3K27me3-BAH domain are independent events and mutually exclusive. The SHL protein could bind to H3K4me3K27me3 peptide with a molar ratio of 1:1 and there is no significant binding affinity difference between singly and doubly modified H3K4me3 and H3K27me3 peptides (Fig. 4). This 1:1 binding ratio indicates that SHL can bind to either H3K4me3 or H3K27me3 separately, but less likely to bind both marks simultaneously. Thus, the dual binding ability of SHL to H3K4me3 and H3K27me3 may function to recruit SHL to either one of these two marks but less likely by a multivalent binding mode.

Flowering is one of the most complex processes in the life cycle of plants, during which some genes are repressed and other genes are activated. Interestingly, many key floral integrators (e.g. SOC1 and FLC) have chromatin states carrying both H3K4me3 and H3K27me3 (Fig. 5f) and their expression is bidirectionally regulated by both H3K4 and H3K27 methyltransferase complexes such as COMPASS and PRC2 and demethylases such as JMJ14 and REF6^50, 51^. The coexistence of the dual marks enables both active and repressive transcription factors to dynamically act on these floral genes to ensure flowering at the appropriate time. A prior study showed that SHL negatively regulates *SOC1* likely via the PHD-H3K4me3 binding and interaction with histone deacetylase 6 (HDA6) to repress flowering^33^. We find that H3K27me3 and CLF are also important for SHL-mediated floral repression. SHL was unable to bind *SOC1* chromatin in the absence of CLF and H3K27me3 (Fig. 6b, c). We further note that SHL-bound genes have much greater overlaps with genes co-marked with H3K27me3 than H3K4me3 (Fig. 5c), consistent with the repressive role of SHL in regulating the flowering integrators. Biochemically, SHL appears to have no preference over the H3K4me3 and H3K27me3 marks with similar binding affinity. It is possible that the local density of H3K4me3 and H3K27me3 may coordinately regulate the chromatin binding affinity of SHL in vivo. As most floral integrators carry both H3K4me3 and H3K27me3 marks, the PHD-H3K4me3 interaction may increase the SHL occupancy at regions with low H3K27me3 enrichment as a backup to ensure the correct floral transition. In some abnormal conditions when H3K27me3 is decreased, the presence of H3K4me3 may also serve as a security protection by recruiting other floral suppressors (e.g. HDA6)^33^ to maintain a repressive chromatin environment. Another possibility is that SHL may recruit PRC2 to establish and maintain an H3K27me3-enriched chromatin environment at its binding regions. Given the unique ability of SHL in recognizing both H3K4me3 and H3K27me3 marks, it will be interesting to investigate whether H3K27me3 and H3K4me3 recognition play a separate or combinatorial role in SHL repression of flowering. Further studies are expected to dissect the precise regulatory mechanism of SHL in chromatin and transcriptional regulation in flowering control.

The unique BAH-PHD dual histone binding module is functionally conserved as demonstrated by similar binding affinity and specificity of Arabidopsis and Populus SHL with H3K27me3 and H3K4me3. Although SHL homology has yet-to-be identified in animals^33^, several chromatin proteins contain both BAH and PHD domains including a mammalian histone methyltransferase ASH1L implicated in leukemia^52, 53^ and yeast transcription factor SNT2 involved in fungi respiration and oxidative stress responses^54^. It is possible that the BAH-PHD cassette of these proteins or other dual domain-containing proteins might similarly recognize both active and repressive marks to balance proper chromatin landscapes to fine-tune the expression of crucial development factors.

## Methods

### Plant materials and growth conditions

The *shl-2* (GT442) allele is in the L*er* background and was a gift from Dr. Manuel Piñeiro (Centro de Biotecnología y Genómica de Plantas, UPM-INIA, Madrid, Spain). The *clf-28* (SALK_139371) and *atx1-2* (SALK_149002), in the Col-0 background, were gifts from Dr. Richard Amasino (UW-Madison). Seeds were sown in soil and kept at 4 °C for two days before transferring to long-day (16 hrs light/ 8 hrs dark) conditions at 22 °C.

### Construction of vectors and generation of transgenic plants

Genomic DNA of SHL with 1kb promoter was amplified and cloned into pENTR/D-TOPO (Invitrogen), recombined into the pEarleyGate302 binary vectors to create an epitope-tagged 3xFLAG fusion (pSHL::SHL-3xFLAG), and transformed into Columbia-0 background. Western blotting and ChIP-seq experiments were conducted using tissues from the homozygous T3 transgenic plants. The wild type AtSHL, BAH (W63L/Y65A), and PHD mutants (F141A/Y148A) were generated in the pSHL::SHL-3×FLAG backbone using a Q5^®^ Site-Directed Mutagenesis kit (New England Biolabs, E0554S) and were transformed into the *shl-2* background via agrobacterium-mediated floral dip method. T1 transgenic plants of Basta resistance were used for leaf counting at the long-day condition. The pSHL::SHL-3xFLAG construct was transformed into *atx1-2* mutants and T1 plants containing the transgenes were used for ChIP-qPCR experiments. The pSHL::SHL-3xFLAG was crossed into a *clf28* mutant and basta resistant F2 plants with *clf28* null mutant background were used for ChIP-qPCR experiments (Supplementary Table 1 for primer information).

### Protein expression and purification

The full-length AtSHL was cloned into a pET-Sumo vector to fuse a hexahistidine-sumo tag to the target protein. The plasmids were transformed into *Escherichia coli* strain BL21(DE3) RIL (Stratagene). The protein expression was induced by adding IPTG to a final concentration of 0.2 mM upon the OD600 of cell culture reaching to 0.7, followed by overnight induction at 20 °C. The recombinant protein was purified using a HisTrap column (GE Healthcare). The hexahistidine-sumo tag was cleaved by ulp1 protease and removed by a second step HisTrap column. The AtSHL protein was further purified using a Superdex G200 column (GE Healcare). The PtSHL was cloned, expressed, and purified using the same strategy as AtSHL. All mutant versions of SHL were generated using a PCR based method and expressed and purified using the same protocol as wild type SHL protein. The peptides were purchased from the GL Biochem Company (Shanghai).

### Peptide microarray assay

Peptide microarray assay was performed as following a previous study^55^. Specifically, the peptide array slide was blocked with blocking solution (1 × PBS, 0.05% Tween-20 pH 7.4, 1% BSA) at 4 °C overnight. After washing three times with 1 ml TTBS buffer (10 mM Tris-HCl, pH 7.5, 150 mM NaCl, 0.05% Tween-20), the slide was incubated with 500 nM GST-tagged recombinant SHL in 1 ml binding buffer (50 mM HEPES, pH 7.5, 50 mM NaCl, 5% glycerol, 0.4% BSA, 2 mM DTT) at RT for 2 h. The slide was then washed with TTBS three times and incubated with an anti-GST antibody (Thermo Fisher, CAB4169, 1:3000 dilution) in TBS with 1% BSA for 1 h at RT. After washing with TTBS three times, the slide was incubated with an anti-rabbit IRDye 650 secondary antibody (LI-COR, P/N 926-65020, 1:5000 dilution) in 1 ml TBS buffer with 1% BSA for 1 h. The slide was washed with TTBS three times, dried by centrifugation, and imaged at dual wavelengths of 532 and 635 nm on Axon GenePix 4000B (Molecular Devices). The laser power was set to 100%, with automatic gain adjustment (0.05% saturation tolerance) for dual photomultipliers. Features in each block were defined by manual adjustment of 13 × 13 grid (feature diameter, 280 μm; column spacing and row spacing, 320 μm) to cover every spot. Signal intensities were quantified by GenePix Pro 6.1 software (Molecular Devices). For each spot, the mean intensities for 635 nm wavelength were used for subsequent analysis. For each peptide species, an average was calculated from three replicate spots. The signal at 532 nm wavelengths was used as a control to identify the misprinting events. Detailed information for peptides on the array can be found in Supplementary Data 1.

### Peptide pulldown assay

The 0.5 nM biotinylated histone peptides were first incubated with 62.5 μl magnetic streptavidin bead slurry (NEB) pre-washed with 1 ml peptide binding buffer (50 mM Tris-HCl, pH 8.0, 300 mM NaCl, 0.1% Nonidet P-40) for 1 h at 4 °C. The peptide-bound beads were washed twice with 1 ml of peptide binding buffer and then incubated with 2 μg GST-tagged SHL proteins in the peptide binding buffer for 3 h at 4 °C. After washing three times with binding buffer, protein-bead complex was boiled in 1XSDS loading buffer (50 mM Tris-HCl, pH 6.8, 2% sodium dodecyl sulfate, 0.01% bromophenol blue, 2 mM dithiothreitol and 2 mM β-mercaptoethanol), subjected to SDS-PAGE gel, and detected with an anti-GST antibody (CAB4169, Thermo Fisher, 1:3000). The raw images of the western blots can be found in Supplementary Fig. 6a.

### Crystallization and structure determination

The PtSHL protein was incubated with the histone peptides with molar ratio of 1:4 at 4 °C for 30 min. The PtSHL in complex with an H3(1-15)K4me3 peptide was crystallized in a condition of 0.2 M potassium acetate and 20% PEG3350. The PtSHL in complex with an H3(20–36)K27me3 peptide was crystallized in a condition of 0.2 M lithium sulfate, 2.0 M ammonium sulfate, and 0.1 M CAPS, pH 10.5. Both crystals were cryo-protected into the reservoir solution supplemented with 15% glycerol and flash cooled into liquid nitrogen. The diffraction data were collected at the beamline BL19U1 of the National Center for Protein Sciences Shanghai (NCPSS) at the Shanghai Synchrotron Radiation Facility (SSRF) and processed using the program HKL2000/3000 package^56^. A summary of the statistics of the data collection for the two complexes is listed in Table 1.

The structure of PtSHL1 in complex with H3K4me3 was solved using the molecular replacement method as implemented in the program Phenix^57^, with the structures of PHD finger of BPTF (PDB code: 2F6N) and BAH domain of PBAF (PDB code: 1W4S) as search models^27, 58^. The manual model building and structure refinement were carried out using the programs Coot and Phenix^57, 59^, respectively. Throughout the refinement, the geometry of structure models was monitored using the program Procheck^60^. The structure PtSHL-H3K27me3 was determined using the same protocol as the PtSHL1-H3K4me3 complex. The statistics of the structural refinement are summarized in Table 1. All the molecular graphics were generated using the program Pymol (DeLano Scientific LLC). The sequence alignment was carried out using the program T-Coffee and illustrated with the ESPript server^61, 62^.

### Isothermal titration calorimetry

The interaction between SHL and histone peptides was monitored on a Microcal PEAQ-ITC instrument (Malvern) at 20 °C. An additional artificial tyrosine residue was added to the C-terminus of the peptide in the ITC experiment for accurately measuring the concentration of peptide by OD280. The protein was dialyzed against a buffer of 100 mM NaCl and 20 mM Tris-HCl, pH 8.0 for 3 h and the lyophilized peptides were dissolved into the same buffer. The titration data were fit using the program Origin 7.0. The titration between AtSHL and H3(1-15)K4me3 peptide yielded serious precipitation, although we tried different pH, salt concentrations, and temperatures.

### Immunoblot analysis

FLAG epitope-tagged proteins were detected with horseradish peroxidase (HRP) conjugated anti-FLAG antibody (Sigma, A8592, 1:1000 dilution). The following histone antibodies were used: H3 (Abcam, ab1791, 1:1000 dilution), H3K4me3 (Millipore, 04-745, 1:1000 dilution), and H3K27me3 (Millipore, 07-449, 1:1000 dilution). All western blots were developed using ECL Plus Western Blotting Detection System (GE healthcare, RPN2132) and chemiluminescent imaging using an Imagequant LAS 4000 (GE healthcare). The raw images of the western blots can be found in Supplementary Fig. 6b–d.

### Chromatin immunoprecipitation

AtSHL ChIP was performed as following the instruction from a previously published study^63^. Two grams above ground tissues of three-week old plants were ground into powder in liquid nitrogen and cross-linked in nuclei isolation buffer I (10 mM Hepes, pH 8.0, 1 M Sucrose, 5 mM KCl, 5 mM MgCl_2_, 5 mM EDTA, 0.6% Triton X-100, 0.4 mM PMSF, and protease inhibitor cocktail tablet (Roche, 14696200)) with 1% formaldehyde for 20 min at room temperature. Crosslinking was stopped with 2 M glycine and the homogenate was filtered through miracloth (Millipore, 475855). Samples were pelleted and re-suspended with 0.3 ml of Nuclear Lysis Buffer (50 mM Tris-HCl, pH 8, 10 mM EDTA, 1% SDS, 0.4 mM PMSF, protease inhibitor cocktail tablet) and 0.7 ml of ChIP Dilution Buffer (1.1% Triton X-100, 1.2 mM EDTA, 16.7 mM Tris-HCl, pH 8, 167 mM NaCl, 0.4 mM PMSF, and protease inhibitor cocktail tablet) before shearing the chromatin by sonication.

For sequential ChIP, the first ChIP with FLAG beads (Sigma, M8823) was performed as described above. For re-ChIP, the DNA-protein complex was eluted twice with 250 μl of 150 ng/μl 3×FLAG peptide (Sigma, F4799) 15 min at RT. The eluted SHL–DNA complex was equally divided into two tubes and incubated with 5 μl of either anti-H3K4me3 (Millipore, 04-745) or anti-H3K27me3 (Millipore, 07-449) antibody for 1 h at 4 °C. The samples were then incubated with pre-washed 40 μl magnetic protein A beads (Thermo Fisher, 10001D) overnight at 4 °C. The beads were washed with low salt buffer (150 mM NaCl, 0.1% SDS, 1% Triton X-100, 2 mM EDTA, 20 mM Tris-HCl, pH 8), high salt buffer (500 mM NaCl, 0.1% SDS, 1% Triton X-100, 2 mM EDTA, 20 mM Tris-HCl, pH 8), LiCl buffer (0.25 M LiCl, 1% NP-40, 1% sodium deoxycholate, 1 mM EDTA, 10 mM Tris-HCl, pH 8), and TE Buffer (10 mM Tris-HCl, pH 8, 1 mM EDTA). The DNA-protein complex was then eluted with Elution Buffer (1% SDS, 0.1 M NaHCO_3_) and reverse cross-linked at 65 °C overnight. After proteinase K and RNase treatment, DNA was purified by the standard phenol–chloroform method and was used for quantitative PCR (Supplementary Table 1 for primer information).

### ChIP-seq and high-throughput sequencing

ChIP-seq libraries were constructed using an Ovation Ultralow DR Multiplex System (NuGEN, #0330) and sequenced using a HiSeq2000 (Illumina) in the UW-Madison Biotechnology Center. Sequencing reads were aligned to the Arabidopsis TAIR10 genome using Bowtie2 (v2.1.0) with default parameters^64^. Reads mapping to identical positions in the genome were collapsed into one read. SICER^65^ was used for peak calling with p = 1e-03. BEDTools (2.17.0) and custom PERL scripts were used for further analysis. ChIP-seq peaks of H3K4me3 and H3K27me3 were obtained from^66^. In SHL genome-wide distribution pattern, log_2_ value of normalized ChIP read density relative to wild-type Col reads was calculated and binned in 100 kb intervals. For plots of H3K4me3 and H3K27me3 level over genes, each gene was divided into 20 intervals (5% each interval) separately for gene body, 2 kb upstream of the transcription start site, and 2 kb downstream of the transcription termination site. All statistical analysis and figures were generated using R (3.2.3). The total reads obtained are listed in Supplementary Table 2.

### Data availability

Coordinates and structure factors have been deposited in the RCSB Protein Data Bank with the accession codes: 5ZNP for PtSHL-H3K4me3 complex and 5ZNR for PtSHL-H3K27me3 complex. ChIP-seq data were deposited into GEO with the accession number GSE111780.

## Electronic supplementary material

Supplementary Information

Description of Additional Supplementary Files

Supplementary Data 1

Supplementary Data 2
