# Supplementary material for: Dual recognition of H3K4me3 and H3K27me3 by a plant histone reader SHL
Source: Nat Commun. 2018 Jun 21;9:2425. doi: 10.1038/s41467-018-04836-y (PMC6013494; doi:10.1038/s41467-018-04836-y)
Supplement: Supplementary file 1 — Supplementary Information [file 41467_2018_4836_MOESM1_ESM.pdf]

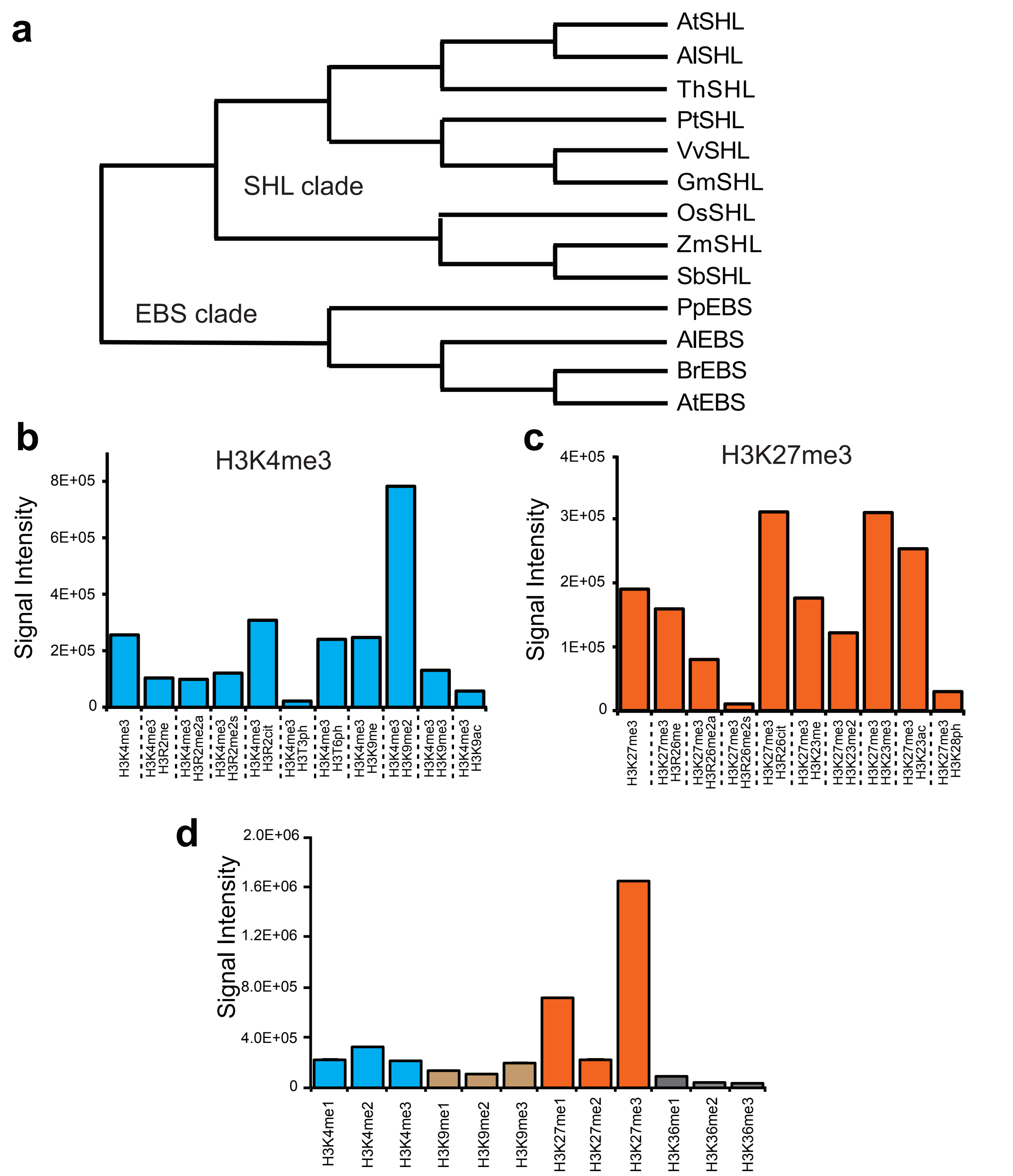

Supplementary Fig. 1 SHL recognizes methylated H3K4 and H3K27 marks. **a** A phylogenetic tree of the SHL and EBS related proteins show that SHL and EBS form independent clade. *Arabidopsis thaliana* (At), *A. lyrata* (Al), *Thellungiella halophila* (Th), *Populus trichocarpa* (Pt), *Vitis vinifera* (Vv), *Glycine max* (Gm), *Oryza sativa* (Os), *Zea mays* (Zm), and *Sorghum bicolor* (Sb). *Physcomitrella patens* (Pp), *Brassica sp* (Br). **b,c** Relative intensity of selective H3K4me3-containing (b) and H3K27me3-containing (d) peptide species. Relative signal intensity is calculated by normalizing each mean signal intensity at 635 nm of triplicate spots to the highest signal on individual subarray, after subtracting background signals (derived from empty spots) for all spots. **d** Relative intensity of SHL binding with different peptides. Peptide species containing same PTMs are grouped together.

a

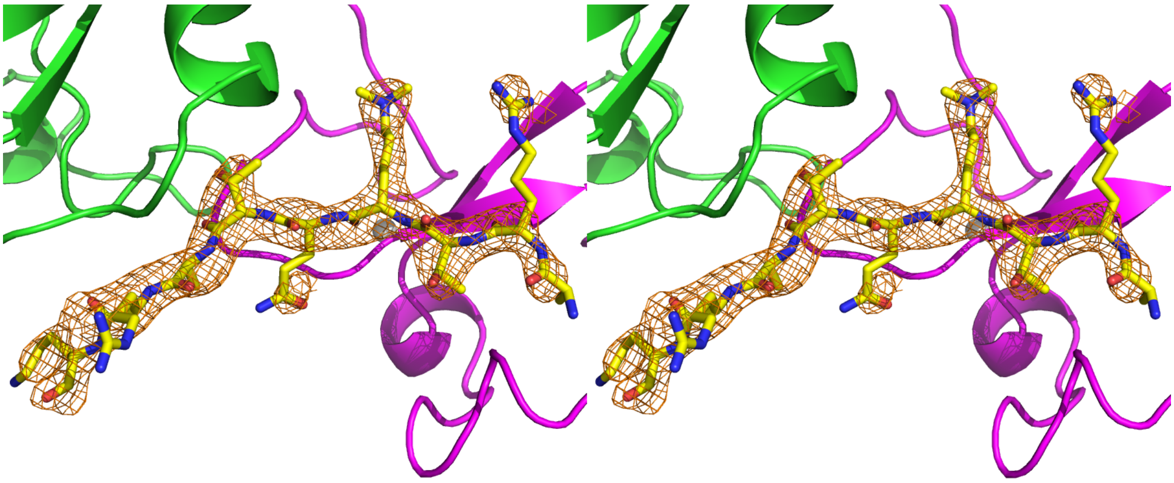

A stereo view of the 2Fo-Fc map of the H3K4me3 peptide

b

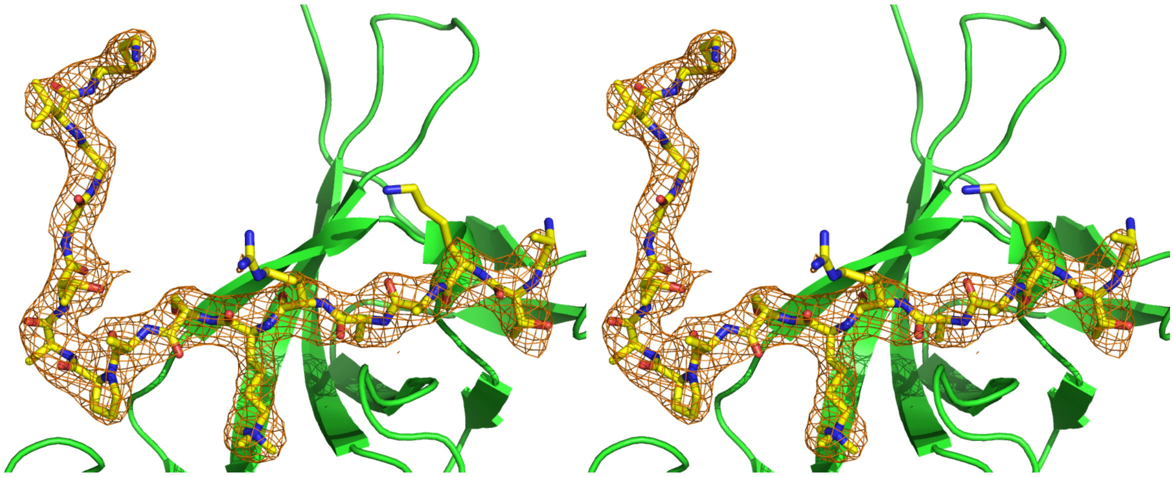

A stereo view of the 2Fo-Fc map of the H3K27me3 peptide

Supplementary Fig. 2 Electron density maps of the peptides. **a** A stereo view of the SIGMAA weighted 2Fo-Fc map of the H3K4me3 peptide at 1 σ level. **b** A stereo view of the SIGMAA weighted 2Fo-Fc map of the H3K27me3 peptide at 1 σ level.

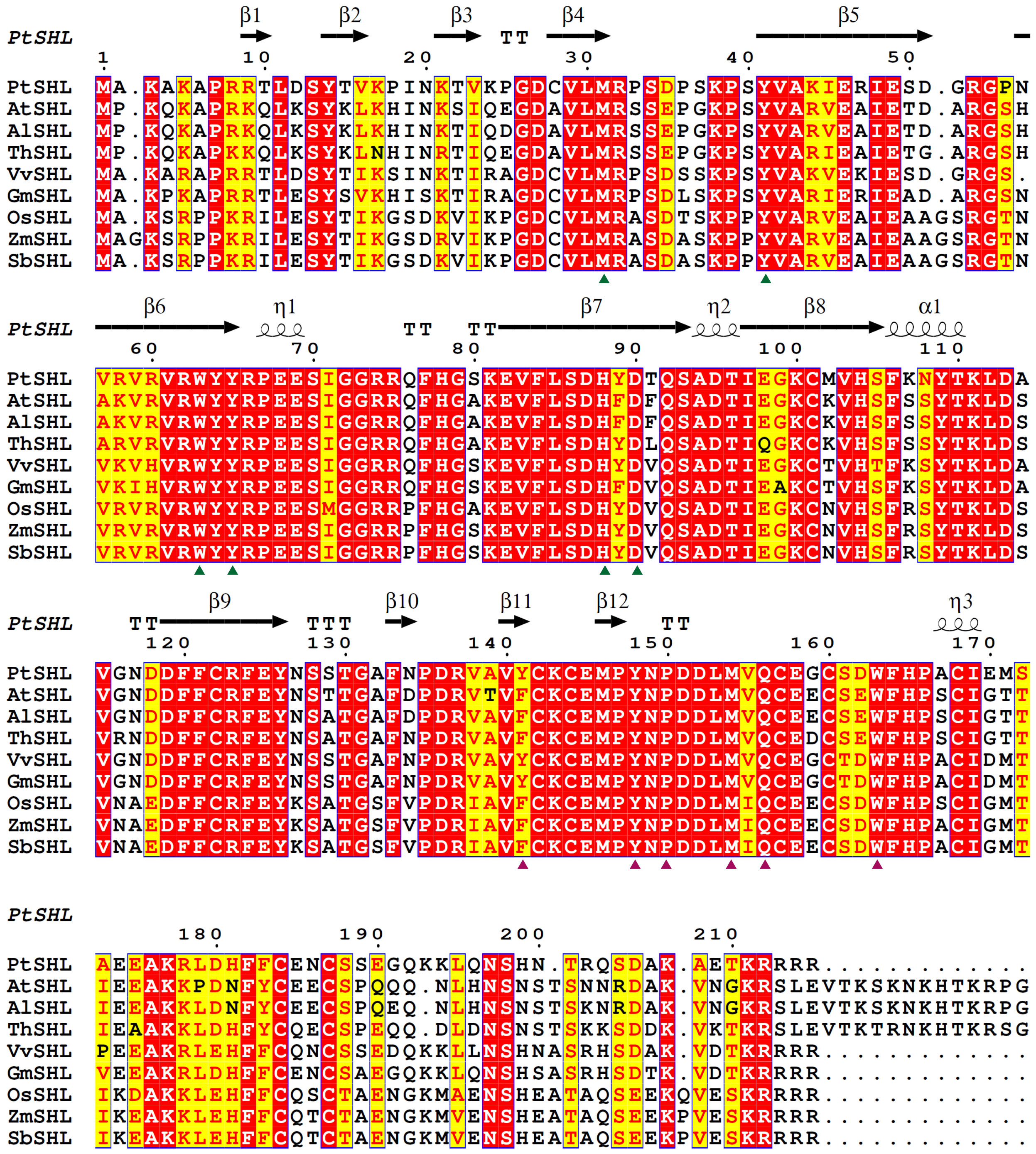

Supplementary Fig. 3 SHL proteins are higly conserved in plants. Sequence alignment of SHL proteins from *Populus trichocarpa* (Pt), *Arabidopsis thaliana* (At), *A. lyrata* (Al), *Thellungiella halophila* (Th), *Vitis vinifera* (Vv), *Glycine max* (Gm), *Oryza sativa* (Os), *Zea mays* (Zm), and *Sorghum bicolor* (Sb). The secondary structures of PtSHL are highlighted on the top of the alignment. The residues involving in H3K27me3-binding and H3K4me3-binding are marked by green and magenta triangles, respectively. All the critical H3K27me3- or H3K4me3-binding residues are conserved.

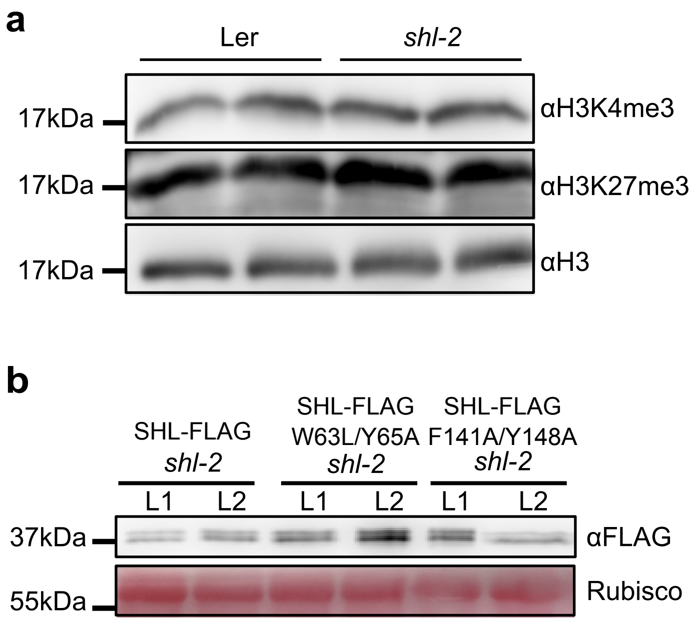

Supplementary Fig. 4 Functional analyses of SHL protein. **a** Western blot analysis of H3K4me3 and H3K27me3 levels in Ler and *shl-2* mutant. H3 serves as a loading control. **b** Western blot analysis of SHL protein levels in *atx1* mutant background using an anti-FLAG antibody. H3 serves as a loading control. **c** Western blot analysis showing SHL protein levels from transgenic plants expressing wild-type SHL-FLAG, H3K27me3 binding defective mutant SHL-FLAG W63L/Y65A, and H3K4me3 binding defective mutant SHL-FLAG F141A/Y148A using an anti-FLAG antibody. Rubisco serves as a loading control. Two independent transgenic lines for each transgene are shown as L1 and L2.

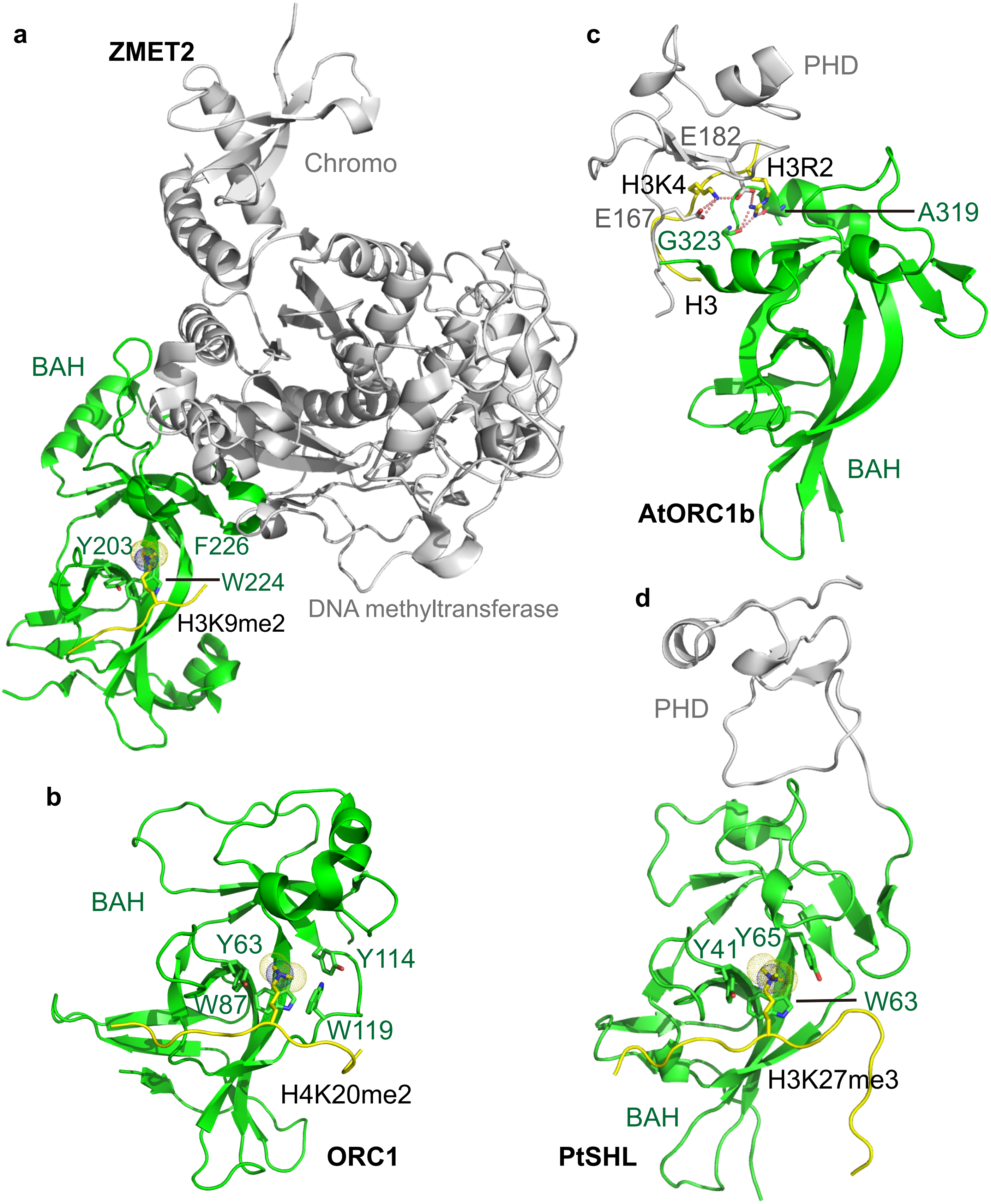

Supplementary Fig. 5 The comparison of the BAH-histone peptide recognition from different structures. (a-d) Structure of maize ZMET2-H3K9me2 complex (a, PDB code: 4FT4), mouse Orc1 BAH-H4K20me2 complex (b, PDB code: 4DOW), Arabidopsis ORC1b BAH-PHD-H3(1-15) complex (c, PDB code: 5HH7), and PtSHL-H3K27me3 complex (d). The BAH domains and the remaining parts of the proteins are colored in green and silver, respectively. The peptide is shown in space-filling representation. The BAH domains are aligned to the same orientation.

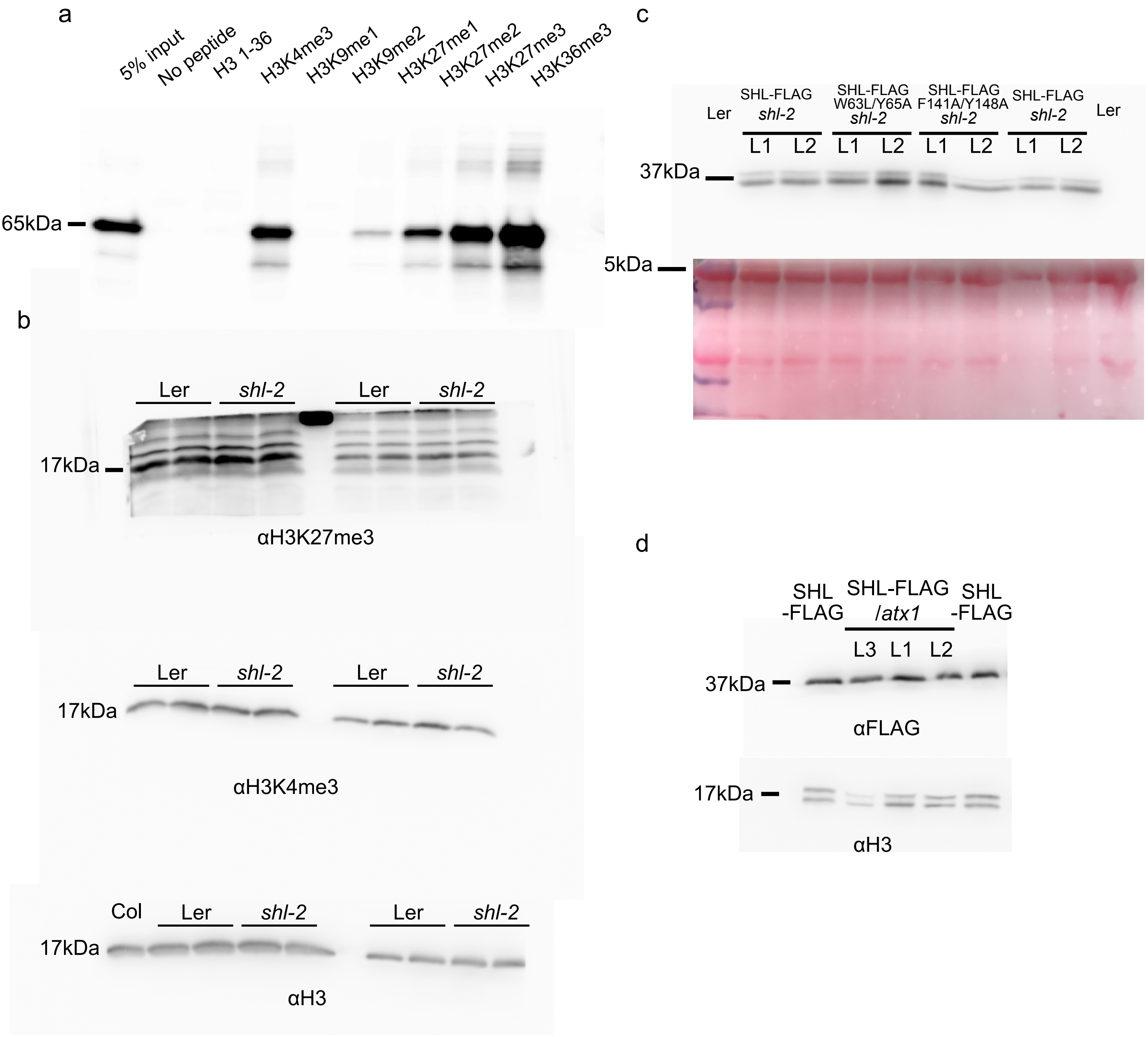

Supplementary Fig. 6 Raw images of western blots in this study
(**a**) Raw images of GST western blot in Fig. 1C. (**b**) Raw image of wstern blot of H3K4me3, H3K27me3 and H3 in Supplementary Fig. 4a. (**c**) Raw images of FLAG western blots in Supplementary Fig. 4b.(**d**) Raw images of FLAG western blot in Supplementary Fig. 4c.

Supplementary Table 1 List of primers used in this study

Name	Primer sequences	Used for
ZP89	caccggatttcattgattatgattgtttg	Cloning to entry vector
ZP90	acctggtcgcttagtgtgtttgttcttc	Cloning to entry vector
ZP57	tgttggaaatcctttcgaatg	Genotype
ZP58	tcatctgggttatacggcatc	Genotype
ZP105	cctggaaaaccgtcggcggtagcgagggtagag	Y41A
ZP106	ctctaccctcgctaccgccgacggttttccagg	Y41A
ZP107	gaaagttcgtgtgaggctgtatgcgcgccctgaggaatctatc	W63L/Y65A
ZP108	gatagattcctcagggcgcgcatacagcctcacacgaactttc	W63L/Y65A
ZP1396	ctgtttgcaggttctgcaagtgtgagatgccgtataacccagatgacttg	F141A/Y148A
ZP1397	caagtcatctgggttatacggcatctcacacttgcagaacctgcaaacag	F141A/Y148A
ZP98	ccgtactccgcgagacatgc	sequencing
ZP20	ggcgtctcgcatatctcattaaagc	sequencing
ZP98	ccgtactccgcgagacatgc	sequencing
ZP22	ggcgtctcgcatatctcattaaagc	sequencing
ZP887	gaagaagatatggtgaggggc	ChIP-qPCR for SOC1
ZP1009	cagcatcacaaagcactgag	ChIP-qPCR for SOC1
ZP230	cgacaagtcaccttctccaaa	ChIP-qPCR for FLC
ZP231	agggggaacaaatgaaaacc	ChIP-qPCR for FLC
ZP1679	cgaagatctcactggcaaatc	ChIP-qPCR for NGA
ZP1680	gaatctccgacacatctttgg	ChIP-qPCR for NGA
ZP7	cgtttcgctttccttagtgttagct	ChIP-qPCR for Actin7
ZP8	agcgaacggatctagagactcaccttg	ChIP-qPCR for Actin7
ZP1297	ctggtcggattaacccaggtg	ChIP-qPCR for EMF1
ZP1298	ggaagatagacgtataaatgaacag	ChIP-qPCR for EMF1
PtSHL	cgggatccatggcaaaagcgaaagcgccgc	Cloning PtSHL
PtSHL	gcggccgcgcggcgctttcgcttttgccat	Cloning PtSHL
AtSHL	cgggatccatgcccaagcaaaaagctccaagg	Cloning AtSHL
AtSHl	gcggccgcttaacctggtcgcttagtgtgtttg	Cloning AtSHL
W163A	cagcgatgcgttccacccgg	PtSHL W163A
W163A	gtggaacgcatcgctgcaacc	PtSHL W163A
Y141A	cgtgttgcggttgcctgtaaatgcg	PtSHL Y141A
Y141A	cgcatttacaggcaaccgcaacacg	PtSHL Y141A
Y148A	gcgaaatgccggccaacccggatg	PtSHL Y148A
Y148A	catccgggttggccggcatttcgc	PtSHL Y148A
M154A	ggatgatctggcggttcagtgcgaag	PtSHL M154A
M154A	gcactgaaccgccagatcatccggg	PtSHL M154A
Q156A	gatggttgcgtgcgaaggttgc	PtSHL Q156A
Q156A	ccttcgcacgcaaccatcagatc	PtSHL Q156A
W63A	gttcgtgcgtactatcgtccggaag	PtSHL W63A
W63A	gatagtacgcacgaacacgaacacg	PtSHL W63A
Y41A	ccgagcgccgttgctaaaatcgaac	PtSHL Y41A
Y41A	gattttagcaacggcgctcggtttag	PtSHL Y41A
Y65A	gttggtacgctcgtccggaagaatc	PtSHL Y65A
Y65A	ccggacgagcgtaccaacgaacacg	PtSHL Y65A

H88A	gtccgatgcctatgatacccagtc	PtSHL H88A
H88A	gggtatcataggcatcggacaggaaaac	PtSHL H88A
D90A	ccgatcactatgccacccagtctgcgg	PtSHL D90A
D90A	gcagactgggtggcatagtgatcggacagg	PtSHL D90A

Supplementary Table 2. Read numbers for ChIP-seq experiments

Sample name	Total raw reads (50bp length)	Reads mapped to genome	Unique mapped reads
Col-0	9,412,540	8,939,183	6,392,775
SHL-FLAG	10,078,136	9,848,692	7,369,668
