# Supplementary material for: Dual recognition of H3K4me3 and H3K27me3 by a plant histone reader SHL
Source: Nat Commun. 2018 Jun 21;9:2425. doi: 10.1038/s41467-018-04836-y (PMC6013494; doi:10.1038/s41467-018-04836-y)
Supplement: Supplementary file 2 — Description of Additional Supplementary Files [file 41467_2018_4836_MOESM2_ESM.pdf]

**Description of Additional Supplementary Files:**

Supplementary Data 1: **Summary of histone peptide arrays.** Quantification of SHL binding intensity on the histone peptide array.

Supplementary Data 2: **Summary of SHL ChIP-seq.** List of genes bound by SHL with P value less than 0.001.
